# Healthcare Professional Advocacy Practices to Promote Patient Safety: A Systematic Review

**DOI:** 10.7759/cureus.105742

**Published:** 2026-03-23

**Authors:** Cecilia Z Kayali, Nadia Baichoo, Natasha D Casas, Kristin M Lefebvre, Kerstin Leuther

**Affiliations:** 1 Clinical Research, University of Jamestown, Fargo, USA

**Keywords:** healthcare professionals, patient advocacy, patient safety, safety culture, systematic review

## Abstract

This systematic review aimed to synthesize qualitative and cross-sectional evidence describing how healthcare professionals (HCPs) apply patient advocacy (PA) practices to support patient safety and to inform strategies that strengthen patient safety through advocacy.

A systematic literature search of CINAHL Complete and EBSCO MegaFILE was conducted on 26 September 2025, and PubMed was searched on 13 March 2026. Qualitative and cross-sectional studies meeting predefined eligibility criteria were included. Affinity mapping was used for thematic analysis, risk of bias was assessed using CASP checklists for qualitative research and for descriptive/cross-sectional studies, and certainty of evidence was assessed with GRADE-CERQual.

Sixteen studies from 13 countries were included. Five interrelated themes described how HCPs advocated for patient safety: interprofessional collaboration, risk prevention, patient education and informed consent, safety-oriented advocacy behaviors, and medication management and polypharmacy. Across studies, advocacy practices involved collaborative decision-making, proactive risk mitigation, patient engagement through education and informed consent, safety-focused clinical prioritization, and vigilant medication management in complex care contexts.

PA for safety is a multidimensional practice expressed through interconnected clinical, relational, and systems-level behaviors across healthcare settings. Recognizing advocacy as a core component of patient safety may guide the development of scalable, context-responsive interventions to support HCPs in safeguarding patients.

## Introduction and background

Medical errors (MEs) have been reported as the third leading cause of death in the United States [1,2]. These MEs can result in psychological and physical harm to patients as well as severe financial loss. About one in 10 patients experiences harm in a primary or ambulatory healthcare setting, and over three million deaths occur annually worldwide due to inadequate provisions for patient safety [3]. The majority (> 50%) of these harmful medical events are preventable, and many are attributed to diagnostic or medication errors, unsafe surgical procedures, care-related infections, patient falls, pressure ulcers, patient misidentification, unsafe blood transfusion, and venous thromboembolism [3]. Globally, MEs may account for trillions of US dollars in costs annually, underscoring the urgent need to make patient safety a priority across all healthcare settings [3].

Sentinel events (SEs), the most severe form of MEs, are safety events unrelated to the patient’s underlying condition that may result in death or serious harm, including permanent or substantial temporary injury [1]. Examples of SEs include wrong surgery, involving the wrong site, procedure, patient, or implant, unintended retention of foreign objects, and delay in treatment [4]. In 2023, the Joint Commission on Accreditation of Health Care Organizations (JCHAO) received 1,411 SE reports [4]. Of all SEs reported, 88% occurred in hospitals, and most were reported voluntarily [4]. The leading SE was patient falls (48%), followed by surgical error and unintended retention of objects (8% each). Surgical errors increased by 26% and foreign objects by 11% from 2022 [4]. Other frequent SEs included treatment delays (6%), fire or burn from medical equipment (4%), and errors in medication management (2%) [4].

Patient safety is a priority across all healthcare settings, encompassing the prevention of harm, reduction of errors, and assurance of high-quality care. Protecting patients from avoidable complications, ensuring effective communication, reducing pain, suffering, and risk of harm during routine and complex care, and minimizing possible financial repercussions remain a focus of all healthcare professionals (HCPs). One strategy for promoting patient safety is through patient advocacy (PA). PA is an ethical, professional responsibility in which HCPs act to safeguard patients’ rights, promote their interests, and support their access to appropriate care, especially in situations where patients may be vulnerable, underserved, or unable to speak for themselves [5]. PA also encompasses the preservation of autonomy, empowering patients to be involved in decisions about their care [6-8].

Optimizing safety protects patients from preventable harm and positively impacts health outcomes and trust with HCPs [3,8,9]. Promoting safety requires a structured framework of organized activities that foster specific behaviors, culture, and environment. This framework encompasses procedures and technologies that minimize risk, prevent avoidable harm to patients, and decrease the occurrence of medical errors [3]. Currently available literature describes the efficacy of communication strategies [10,11], but provides a limited understanding of the relationship between HCPs’ specific PA practices and their impact on patient safety.

Promoting PA as a culture may positively impact safety and help prevent avoidable SEs and MEs within organizations [1,12]. However, time limitations and job pressures may influence HCPs’ ability to provide effective advocacy [13]. As a result, patients may receive lower quality care resulting in poorer outcomes [12,13]. HCPs must advocate for their patients to promote high-quality care and minimize preventable errors. This systematic review aims to synthesize existing evidence on current PA practices to inform strategies for improving patient safety. Despite growing recognition of PA as a critical determinant of safety, how advocacy practices are enacted across HCP roles and clinical settings remains poorly defined. Existing research often explores advocacy conceptually or within specific disciplines, but it rarely examines the specific practices through which HCPs advocate for patient safety across diverse clinical settings and professional roles. Current literature lacks an integrated synthesis across HCP roles and settings, leading to a fragmented understanding of how advocacy practices can be operationalized and sustained at a systems-level. By addressing this gap and examining the literature for patterns of advocacy behaviors that demonstrably promote patient safety, this systematic review and thematic synthesis seek to identify evidence-based strategies that enable HCPs to enhance the effectiveness of their advocacy practices to facilitate patient safety.

## Review

Methods

This systematic review and thematic synthesis evaluated peer-reviewed published literature that included PA practices of HCPs in different healthcare settings worldwide. The review followed guidelines from the Preferred Reporting Items for Systematic Reviews and Meta-Analyses (PRISMA) [14], and the PRISMA in Exercise, Rehabilitation, Sport medicine and SporTs science (PERSiST) [15]. The review was registered on PROSPERO (CRD42024618794).

Study Eligibility

Eligible studies investigated the attributes, perceptions, and behaviors of HCPs who advocated for patient safety across qualitative and cross-sectional study designs. Studies evaluated physicians and/or nurses of any specialty and healthcare setting. No age restrictions were applied to study participants; some included studies involved patient participants of varying ages. Studies identified PA practices that facilitated patient safety in hospitals, nursing homes, or long-term care clinics. Studies were excluded if the participant characteristics specifically involved other health professions such as pharmacists, radiologists, dentists, or technicians. Interventions using artificial intelligence (AI) or simulation, mobile apps, telehealth, or training programs were also excluded.

Search Strategy

A university librarian assisted in developing search terms as seen in Table 1. CINAHL Complete, and EBSCO MegaFILE databases were searched on 26 September 2025, and a 'peer-reviewed' filter was applied. The PubMed database search was performed on 13 March 2026 using the librarian-developed search string with no filters applied. No limits were placed on publication year, language, or study type.

**Table 1:** Search terms

Database	Strategy
EBSCO: CINAHL Complete; EBSCO MegaFILE	("patient safety" OR “clinical safety”) AND ("patient advocacy" OR "patient advocate" OR “healthcare advocacy” OR “healthcare advocate") AND (ethics OR "ethical behavior" OR "ethical attitudes" OR "ethical standards") AND ("healthcare professionals" OR “healthcare providers” OR "health care professionals") Filter – peer-reviewed
PubMed	("patient safety" OR “clinical safety”) AND ("patient advocacy" OR "patient advocate" OR “healthcare advocacy” OR “healthcare advocate") AND (ethics OR "ethical behavior" OR "ethical attitudes" OR "ethical standards") AND ("healthcare professionals" OR “healthcare providers” OR "health care professionals") Filter – none

Study Selection

At least two reviewers independently screened 937 articles identified in the searches. Duplicates and conference abstracts were excluded prior to screening. Titles and abstracts were reviewed for relevance and excluded if the following criteria were met: irrelevant topic, presentation abstract, editorial, or no English translation. Two additional records were identified through supplementary searching conducted prior to the formal librarian-assisted database search. These records were assessed using the same predefined eligibility criteria applied to all database-retrieved records. Duplicate checking was conducted against the full set of database-identified records using title, author, and DOI verification. Both studies were confirmed as peer-reviewed publications and met all inclusion criteria prior to inclusion. 

Data Collection

Data extraction from eligible studies was supported by structured automation tools to improve efficiency and reduce transcription errors, while interpretive judgment and final data decisions were made by the first author. Data extraction for studies identified in the original search was assisted using Afforai AI Research Assistant and Elicit [16,17]. Data extraction for studies identified in the updated search was supported using Logically (formerly Afforai) and Elicit [17,18]. Data extraction for the additionally identified study was supported using Logically [18]. Across all search phases, these automation tools were used solely to facilitate information retrieval and organization; the authors retained full responsibility for data verification, inclusion decision, and interpretation. Extracted variables included author, year, study design, participants (sample size, age, sex), setting, and qualitative outcome.

Quality Assessment

Risk of bias was assessed with Critical Appraisal Skills Programme (CASP) checklist for Qualitative Research [19], and CASP checklist for Descriptive/Cross-Sectional Studies [20].

Outcomes and Analysis

The primary qualitative outcome was the identification of advocacy practices of HCPs to enhance patient safety. Key themes across studies were synthesized using affinity mapping, in which each relevant finding was grouped according to thematic relatedness. ChatGPT (GPT-5, OpenAI, 2025) was used to assist with consolidating and drafting narrative summaries of author-derived themes following thematic grouping. The first author independently identified subthemes pertaining to the research question across all included studies, organized them into five major thematic groupings, and color-coded subthemes by thematic similarity prior to AI-assisted consolidation. ChatGPT was used to generalize the grouped subthemes into consolidated summary statements, which the first author cross-checked against the original groupings to ensure accurate representation. For the additionally identified study, subthemes were independently identified and grouped according to the existing major themes, and integrated into the synthesis with support from Claude (Anthropic, 2026), with adjustments made to affected thematic items as needed. All thematic synthesis, interpretation, and final analytic decisions were conducted by the authors.

Certainty of Evidence

Confidence in qualitative and cross-sectional findings was assessed using the Confidence in the Evidence from Reviews of Qualitative Research (GRADE-CERQual), considering methodological limitations, coherence, adequacy of data, and relevance [21]. Domains were graded according to the level of concern about their potential impact on confidence in review findings.

Results

Search Results

**Figure 1:**
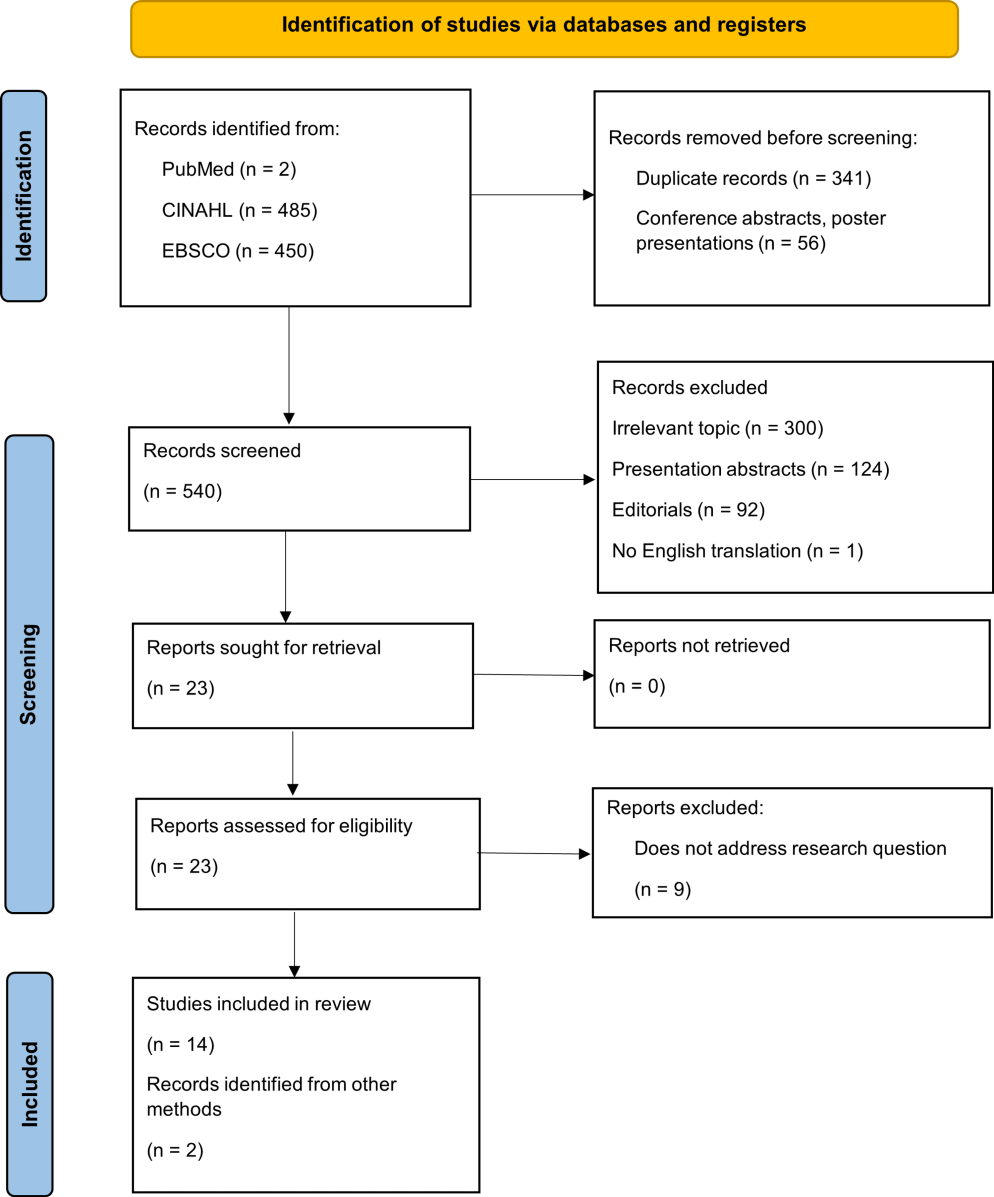
PRISMA flow diagram PRISMA: Preferred Reporting Items for Systematic Reviews and Meta-Analyses

Database searches identified 937 articles, 540 of which were screened after removing 397 duplicates, conference abstracts, and poster presentations. Per eligibility criteria, 517 articles were excluded following review of titles and abstracts. Full text was assessed in 23 articles, 14 of which met eligibility criteria, while nine did not address the research question. Along with two records identified through supplementary searching, the review included 16 studies (Figure 1).

Characteristics of Included Studies

The final analysis included 14 qualitative research studies [22-35] and two cross-sectional studies [36,37]. Sample size ranged from six to 244 participants. All studies involved nurses; four studies also included physicians. The studies were conducted in 13 countries including the United States [22], Sweden [23,32], Japan [24], Australia [25], Ghana [26], United Kingdom [27], Saudi Arabia [28], South Africa [29], Iran [30,33], China [31,37], Italy [34], the Netherlands [35], and Nigeria [36].

Qualitative studies used semi-structured interviews, naïve sketches, focus groups, non-participant observations, and shadowed observations with contextual inquiries [22-35]. Cross-sectional studies used self-reported questionnaires with or without narrative responses, and examined HCPs’ knowledge, practice of advocacy roles, and self-assessed performance across CARE model dimensions (competence, altruism, responsibility, empathy) [36,37]. Settings across all studies included inpatient hospitals (tertiary, acute, pediatric, university), surgical and ambulatory wards, home nursing, and chronic disease care facilities. Study characteristics are summarized in Table 2.

**Table 2:** Summary of reviewed studies and characteristics SD: Standard deviation; yo: years old; US: United States; yrs: years; HCPs: healthcare professionals; CVD: cardiovascular disease; PCC: person-centered care, patient-centered care; CIED: cardiac implantable electronic devices; DSH: deliberate self-harm; OR: operating room

Author, year	Study Design	Total (#) Participants	# of Female vs Male Participants	Age of Participants (SD)	Setting	Qualitative Outcomes
Paynton (2009) [22]	Qualitative study using narrative analysis and grounded theory approaches, involving interviews over a six-month period	6 registered nurses	Gender breakdown not specified	average age = 43 yo	A variety of hospitals with different clinical populations (US)	Focused on how nurses utilize informal power strategies to advocate for patient care, revealing various themes from the narratives of six registered nurses. Outcomes included insights into how nurses managed organizational and hierarchical constraints to influence patient care positively. Key strategies: circumnavigation of organizational constraints, direct confrontation with physicians and the doctor-nurse game, where nurses convey critical information indirectly (the "game") to avoid direct confrontation with doctors.
Andersson et al. (2015) [23]	Qualitative, phenomenographic study using a purposive sampling approach, involving interviews and thematic analysis	21 registered nurses	20 females, 1 male	age range: 23-63	2 county hospitals (Sweden)	Identified descriptive categories that reflect how nurses understand and conceptualize caring: person-centeredness, safeguarding the patient's best interests, nursing interventions, contextually intertwined. Patient-centered care: emphasized the importance of understanding the patient's context and individual needs in providing effective care.
Toda et al. (2015) [24]	Qualitative study using semi-structured interviews	21 psychiatric nurses	15 females, 6 males	mean age: 44.5 yo (SD=7.5 yrs)	Various facilities including psychiatric hospitals and home-visit nursing stations (Japan)	Focused on the judgments made by psychiatric nurses regarding when to intervene as patient advocates. Key findings: impediments to patient safety; barriers to patient decision-making; inappropriate treatment or care.
Chaboyer et al. (2016) [25]	Qualitative, multi-site, collective case study approach with semi-structured individual and group interviews using purposive sampling	62 (patients, families, HCPs (physicians/nurses), and volunteers/ administrators	Gender breakdown not specified	Age range/average not specified	5 private and public hospitals (Australia)	Explored how HCPs engaged patients in communication during care transitions. Themes identified: organizational commitment to patient engagement; influence of hierarchical culture on patient engagement; understanding and negotiating patient preferences.
Dadzie et al. (2017) [26]	Exploratory descriptive qualitative study using purposive sampling and semi-structured interviews	15 nurses	14 females, 1 male	age range: 25-55 yo and older	1 hospital (Ghana)	Explored characteristics that influence nurses' advocacy roles, including moral courage, empathy, communication skills, and assertiveness.
Samuriwo et al. (2021) [27]	Qualitative narrative study using semi-structured interviews	20 nurses (ward-based and in extended roles)	18 females, 2 males	age range not specified average yrs of experience: 21 yrs	19 acute patient care hospitals (Wales, UK)	Focused on how nurses construct their identities in relation to their interactions with trainee doctors regarding patient safety. Identities identified: teacher; guardian of patient well-being; provider of emotional support; navigator; team player.
Alodhialah et al. (2024) [28]	Qualitative descriptive study using semi-structured interviews	15 registered nurses (caring for older adults with multimorbidities)	gender breakdown not specified	age range: 26 and 55 yo	Various healthcare facilities with high volumes of adult patients (Riyadh region, Saudi Arabia)	Aimed to identify ethical and legal challenges faced by nurses in caring for older adults with multimorbidities. Key themes: patient autonomy and informed consent; management of polypharmacy; end-of-life care and advance directives.
Lesao et al. (2024) [29]	Qualitative, exploratory, descriptive study using naïve sketches for data collection	8 professional nurses	8 females, 0 males	18-40 yo	hospital settings with neonates, infants, and children under-5 (South Africa)	Eight identified categories of nurses' professionalism attributes that influence quality of care for neonates, infants, and children under 5: Knowledge, spirit of inquiry, accountability, autonomy, advocacy, collegiality and collaboration, ethics and values, and professional reputation. Each category generated sub-themes that further describe the specific aspects of professionalism that contribute to quality care.
Nabi Foodani et al. (2024) [30]	Qualitative study using qualitative content analysis of semi-structured, face-to-face interviews	23 nurses with chronic cardiovascular disease	16 females, 7 males,	age range: 26-62; average age=45.6 yo (SD=10.9)	No details on the type of healthcare facility. (Tehran, Iran)	Explored the care needs of patients with chronic CVD from the perspective of nurses with CVD. It emphasized the importance of a holistic, patient-centered approach that prioritizes effective communication, seamless access to care, and continuity of post-discharge follow-up. Themes identified: receiving care from a responsive system, capacity building for the patient, receiving multidimensional care, having a comprehensive support system, and utilizing new caregiving technologies. Empowering patients through education and developing supportive systems, including access to multidisciplinary teams and community resources, is crucial for effective care.
Zhou et al. (2024) [31]	Descriptive phenomenology qualitative design using semi-structured and in-depth interviews	18 CIED patients and 20 HCPs (including 13 physicians and 7 nurses) involved in CIED surgeries	Patients: 7 females, 11 males HCPs: 9 females, 11 males	patients: between 19 and 88 yo HCPs: age range not specified	1 tertiary hospital (Yunnan Province, China)	The study aimed to explore and analyze the intraoperative care experiences of patients and HCPs during CIED surgery. 4 themes identified: safety and success as a priority; humanistic care is essential yet often lacking; the paradox of surgery information given; suggestions for improving surgery experience.
Löfström et al. (2025) [32]	Qualitative descriptive, observational study using semi-structured interviews	10 nurses, 8 psychiatrists	Nurses: 5 females, 5 males Psychiatrists: 3 females, 5 males	age range/average not specified	Psychiatric clinic (Sweden)	Explored patients' and HCPs' perceptions of responsibility and autonomy in psychiatric hospital care for patients with DSH. It revealed that psychiatrists focus on legal aspects and nurses focus on ethical aspects. Adapting to other professionals' opinions can decrease autonomy, but can increase responsibility. Renunciation of autonomy to achieve cooperation is considered important for effective interprofessional collaboration.
Mousazadeh et al. (2025) [33]	Descriptive qualitative content analysis of semi-structured interviews	16 nurses	Predominantly female, gender breakdown not specified	age range: 27-52 yo (mean=38)	Public teaching hospitals (Northern Iran)	Found a dichotomous perspective among nurses regarding PCC. Some participants viewed PCC as essential for quality care, while others viewed it as an unnecessary luxury. The main theme of 'luxurious or necessary' underlines the categories of 'luxurious care' and 'enriched nursing care'. A framework is provided for implementing PCC that respects patients' dignity and autonomy while acknowledging the challenges nurses face in delivering such care.
Reato et al. (2025) [34]	Qualitative descriptive study with shadowed observations supplemented with brief, contextual inquiries	6 nurse managers, 12 staff nurses, 28 postgraduate nursing students	42 females, 4 males	Mean age=36 yo (SD=12.4)	Various hospitals (Northern Italy)	Identified 15 transversal competencies, 50 sub-competencies, and 153 specific tasks and activities for OR nurses. Integrating these competencies in healthcare is critical for patient safety, team performance, and high-quality care in complex surgical environments.
Van Mersbergen-de Bruin et al. (2025) [35]	Qualitative study using non-participant observations, semi-structured interviews, and focus groups (mono- and multidisciplinary)	35 nurses, 4 head nurses, 1 manager, 2 nurse practitioners, 1 team lead	38 females, 5 males	Age range: <21->60	Surgical and ambulatory wards in an urban, 600-bed Dutch teaching hospital (the Netherlands)	Qualitative thematic outcomes addressing challenges nurses face and the situated resilience strategies they employ to safeguard care quality and safety (e.g., compliance tactics, investigative approaches, relational negotiation, alliance-building).
Kolawole (2017) [36]	A cross-sectional observational study that used a combination of self-report questionnaires from nurses and in-depth interviews for patients	219 nurses, 25 surgical patients	Nurses: 190 females, 29 males: gender breakdown not specified	Nurses' age range: 21-60 yo patients' age range: 20 yo or younger - 50 yo or older	3 (Federal, State, and Private) healthcare facilities (Nigeria)	Assessed nurses' knowledge about their advocacy roles and how this knowledge translated into practice.
Fang et al. (2020) [37]	Cross-sectional survey study including a survey with both narrative responses and a 4-point rating scale	157 nurses	155 females, 2 males,	age range: 21-55 yo (SD=6.06)	Internal medicine department of a university-affiliated hospital (China)	Assessed nurses' perceptions based on dimensions of the CARE model: competence, altruism, responsibility, and empathy. Nurses rated their performance on the dimensions (1=very poor to 4 very good). Nurses rated themselves as 'good' to 'very good': Competence: 68.79%; Altruism: 73.25%; Responsibility: 86.62%; Empathy: 81.53%

Study Quality Assessment

Risk of bias was assessed as low among qualitative studies. Eleven studies were considered to have no concerns [23,25-32,34,35], while two had minor issues, including not reporting future research needs [22], and unclear statement of study’s aim [33]. One study had moderate concerns due to limited transparency in data saturation, data analysis, transferability, and reporting rigor [24]. Most qualitative studies clearly stated research aims, and all used appropriate methods, considered ethics, and reported findings.

**Figure 2:**
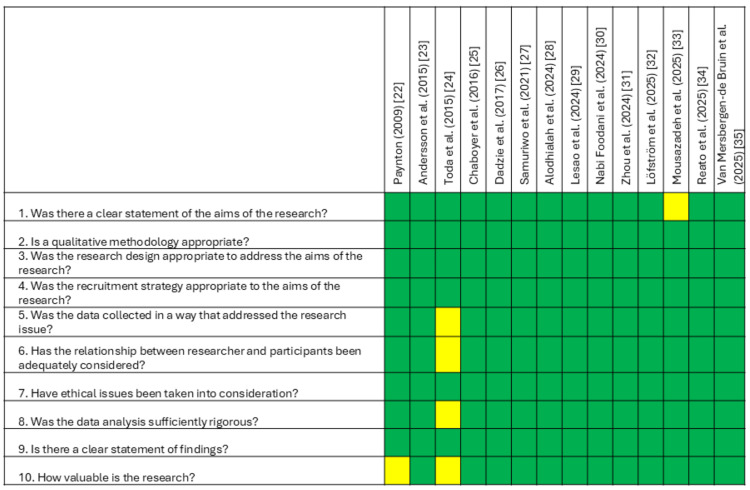
Risk of bias for qualitative studies CASP checklist for qualitative research Green: yes; Yellow: can't tell; Red: no

**Figure 3:**
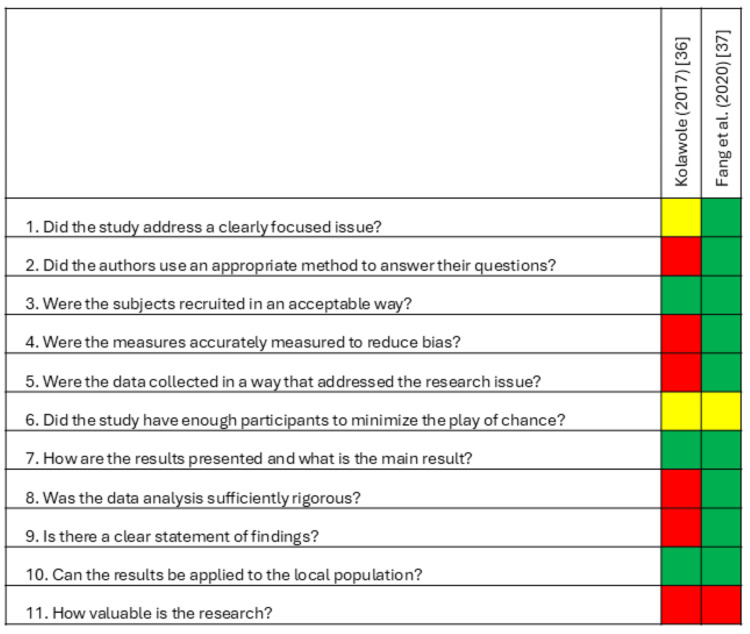
Risk of bias for cross-sectional studies CASP checklist for descriptive/cross-sectional studies Green: yes; Yellow: can't tell; Red: no

Among cross-sectional studies, one was rated low risk, though it lacked a reported power analysis [37]. The other study had high risk of bias due to insufficient methodological detail, incomplete reporting of results, unclear data analysis rigor, and lack of generalizability [36]. Summaries of risk of bias assessments are presented in Figure 2 (qualitative) and Figure 3 (cross-sectional).

Thematic Synthesis

Five interrelated themes were identified that describe how PA is enacted by HCPs to promote patient safety: interprofessional collaboration (12/16 studies), advocacy through risk prevention (11/16 studies), advocacy through education and informed consent (10/16 studies), safety-oriented advocacy (6/16 studies), and advocacy in medication management and polypharmacy (3/16 studies). Table 3 summarizes the key themes.

**Table 3:** Summary of thematic synthesis PCC: person-centered care, patient-centered care; HCPs: healthcare professionals

How do HCPs advocate for their patients to promote safety?	
Key Themes	References
Interprofessional Collaboration in Patient Advocacy	
Proactively exercising collective clinical discretion & challenging institutional constraints & conflicting stakeholder demands to avert unnecessary patient harm.	[22,24,35]
Proactively engaging in strategic communication and expert-informed influence, and seeking allies or second opinions when feeling unheard, to guide collaborative clinical decision-making and orient colleagues to essential ward resources, ensuring patient-centered and safety-driven outcomes.	[22,26,27,32,35]
Acting as intermediaries between patients and the treatment team to effectively communicate patients' condition status & preferences & create a personalized discharge plan based on the patient's needs.	[23,27,30,35,36]
Gathering patient-specific information & voicing ethical or clinical concerns in team meetings to support open dialog, collaborative risk identification, & informed adjustments to care decisions.	[24,26,27,28,32,34,35]
Promoting inclusive collaboration through mutual respect, conflict resolution, & self-awareness of one's role & impact within the team.	[32,34,35]
Using clear documentation and digital tools, including targeted action-oriented notes, to support multidisciplinary communication, align care plan expectations, and ensure effective continuity of care.	[28,29,30,32,34,35]
Advocacy Through Risk Prevention	
Continuously pursuing advanced healthcare education and training and complying with and reinforcing regulations and policies among colleagues to deliver safe, quality care.	[29,30,32,33,34,35]
Protecting patient rights, clearly defining problems by identifying and interpreting key information, voicing treatment-related concerns, and exploring solutions strategically by being active listeners.	[29,30,32,34,35,36]
Involving patients in their treatment's decision-making to provide empathetic, compassionate, evidence-based PCC that is personalized based on each patient's situation & preferences.	[30,33,37]
Collecting vital patient data and responsibly using their own judgment to balance between patient autonomy & lead HCPs' clinical recommendations to prioritize safety.	[23,27,28,32,35]
Creating detailed care documentation to form accurate diagnoses, tracking clinical procedures to anticipate clinical challenges, initiating discharge planning early, and establishing a post-discharge care framework to provide continuous support.	[23,30,35]
Focusing on evidence-based results, vigilantly observing their environment, situations, and signals from patients and colleagues, proactively identifying risks, complications, and resource needs, and quickly addressing patient needs to improve their symptoms and comfort.	[23,32,34,35,37]
Prioritizing their own mental & physical health to help remain calm, focused under pressure, capable of making quick, ethical, evidence-based decisions, respond appropriately to unsafe practices, exchange constructive feedback while promoting integrity, accountability, & legal compliance.	[33,34]
Patient Advocacy Through Education and Informed Consent	
Using transparent, customized communication with patients & families, ensuring understanding of treatment, medication adherence & interactions, & care plans to prevent risks & re-hospitalization.	[23,24,28,35]
Educating & supporting patients on necessary medical procedures, possible risks, alternative methods, & manage fears, actively participating in care decisions.	[28,30,31,35]
Empowering & encouraging patients through targeted education on treatment plans & options to promote patient involvement & safety while respecting patient rights.	[25,26,35,36]
Equipping patients with health literacy through personalized routine-based simulations on self-care & management to ensure autonomy, continuity of care & compliance at home.	[24,29,30]
Safety-Oriented Patient Advocacy	
Prioritizing safety-critical tasks & successful clinical outcomes over patient comfort or non-urgent care when necessary to ensure accurate & safe treatment.	[23,31,35,37]
Triaging patients to identify & attend to patients most at risk, ensuring timely & safe care delivery.	[26,35]
Remaining attentive to patients' pain & emotional distress, prioritizing actions to alleviate suffering.	[33,35]
Advocacy in Medication Management and Polypharmacy	
Educating patients & caregivers about their medications, emphasizing adherence, potential risks, & maintaining accurate medication lists.	[26,28]
Vigilantly managing complex medication regimens through monitoring, regular reviews, accurate documentation, & interprofessional communication to prevent adverse drug interactions & adjust treatment as needed.	[28,35]

Interprofessional Collaboration in Patient Advocacy

Twelve studies emphasized interprofessional collaboration as central to PA and safety [22-24,26-30,32,34-36]. HCPs described questioning constraints and navigating competing demands, engaging in strategic communication and expert-informed influence, and seeking allies or second opinions when feeling unheard, to guide collaborative clinical decision-making. HCPs acted as intermediaries between patients and colleagues, assessed patient conditions, and voiced safety concerns within multidisciplinary teams. HCPs also highlighted the use of clinical documentation systems, including targeted action-oriented notes, to promote alignment of care plans and continuity across disciplines, recognizing that individual actions and professional roles influenced overall team performance and patient outcomes.

Advocacy Through Risk Prevention

Eleven studies described PA as enacted through proactive risk prevention strategies [23,27-30,32-37]. HCPs emphasized continuous health education, adherence to standards of care and reinforcing policies among colleagues, identification of treatment-related problems, and exploration of corrective solutions. Advocacy was expressed through the provision of empathic, evidence-based person-centered care, which supported early recognition of patient needs and potential safety risks. HCPs applied clinical judgment to prioritize safety, thoroughly assessed and documented diagnoses, tracked clinical procedures to anticipate challenges, and proactively developed post-discharge care plans. HCPs also reported maintaining situational awareness to proactively identify and address emerging clinical risks. They also recognized that supporting clinician mental and physical well-being is essential for maintaining composure, focus, and patient safety during high-pressure care delivery.

Patient Advocacy Through Education and Informed Consent

Ten studies showed how HCPs advocated for safety through patient education and informed consent [23-26,28-31,35,36]. HCPs emphasized clear and inclusive communication with patients and all stakeholders regarding treatment plans, risks associated with treatment interruption or discontinuation, and potential medication interactions. HCPs highlighted exploring alternative administration methods, addressing patients’ fears and concerns, and ensuring patients understood available treatment options. They empowered patients to actively participate in care decisions by providing targeted education, and training for at-home care, including personalized simulations to support understanding and adherence.

Safety-Oriented Patient Advocacy

Six studies described HCPs’ advocacy through safety-oriented clinical decision-making [23,26,31,33,35,37]. HCPs triaged patients to identify those requiring immediate attention, and prioritized safety-critical tasks to ensure accurate and safe care delivery. HCPs prioritized successful clinical outcomes, even when this involved trade-offs with patient comfort. HCPs also reported remaining attentive to patients’ pain and suffering, and prioritizing actions aimed at alleviating physical and emotional distress.

Advocacy in Medication Management and Polypharmacy

Three studies addressed advocacy in polypharmacy management [26,28,35]. Strategies included educating patients on proper medication administration and the risks of dose interruptions, conducting regular medication reviews, and coordinating with other HCPs to clarify and reconcile prescriptions. HCPs remained vigilant and shared responsibility in managing complex medication regimens among patients with multiple chronic conditions to prevent complications and ensure safe treatment.

Certainty of Evidence

Based on GRADE-CERQual assessment, the certainty of evidence ranged from low to moderate. Review findings with moderate confidence included polypharmacy and safety-oriented PA, while interprofessional collaboration, patient education and informed consent, and risk prevention were assessed as low confidence. A summary of the confidence assessment is provided in Table 4.

**Table 4:** Summary of the CERQual confidence assessment GRADE-CERQual: Confidence in the evidence from reviews of qualitative research; HCPs: healthcare professionals; PCC: person-centered care, patient-centered care

Review Findings	Studies Contributing to the Review Finding	Overall CERQual Assessment of Confidence	Explanation of Judgment
Interprofessional Collaboration: HCPs advocated for their patients to promote safety by voicing concerns and disagreements, asking questions about patients' status, acting as intermediaries between patients & treatment team, gathering patient-specific information to support collaborative decision-making, & maintaining thorough documentation to ensure all parties are well informed	[22,23,24,26,27,28,29,30,32,34,35,36]	Low confidence	2 studies with methodological limitations: 1 with minor concerns (data saturation not stated, vague data analysis rigor & researchers' role in data collection, analysis & selection, lacks generalizability), 1 with serious concerns (limited description of instruments used & results). 3 studies with minor concerns about relevance (limited generalizability, results lack evidence of collected data). 1 study with serious concerns about coherence and moderate concerns about adequacy (lacks details of the type of questions used to collect data, incomplete results).
Risk Prevention: HCPs vigilantly evaluated treatment & identified risks, addressed unethical/unsafe practices, took accountability for their decisions, proactively worked on discharge planning, and questioned unnecessary procedures. HCPs pursued advanced education/training, adhered to standards & guidelines, monitored patients to alleviate symptoms, balanced patient autonomy with safety when choices posed danger, and provided evidence-based PCC.	[23,27,28,29,30,32,33,34,35,36,37]	Low confidence	3 studies with methodological limitations: 2 with minor concerns (unclear study aim, no power calculation, lack of generalizability), 1 with serious concerns (does not address clearly focused issue, vague data analysis rigor, unclear statement of findings, limited description of instruments used & results). 2 studies with minor concerns about relevance (no power calculation, results lack evidence of collected data). 1 study with minor concerns about coherence and moderate concerns about adequacy (vague details of questions used to collect data, rigor of data analysis, incomplete results).
Patient Education/Informed Consent: HCPs promote informed, shared decision-making, explain condition status & treatment options, procedures & possible risks, offer education to support post-discharge care.	[23,24,25,26,28,29,30,31,35,36]	Low confidence	2 studies with moderate methodological limitations (lack details on data saturation/power calculation, limited description rigor of data analysis, used instrument, & results). 2 studies with minor concerns about relevance, coherence and adequacy (results lack evidence of collected data, lack details of the type of questions used to collect data, rigor of data analysis, incomplete results).
Safety-Oriented PA: HCPs triage patients to prioritize care, prioritize surgical success over short-term comfort, and prioritize essential patient care to alleviate pain & suffering.	[23,26,31,33,35,37]	Moderate confidence	2 studies with low methodological limitations (lacking details on power calculations, no clear statement of study aims), 1 study with minor relevance concerns about limited generalizability. No or very minor concerns about coherence and adequacy.
Polypharmacy: HCPs frequently review, assess, & adjust medication regimens to prevent drug interaction risks, explain medication administration, risks, & interruption consequences, counsel on the importance of maintaining medication lists, & gather medication clarifications from other HCPs	[26,28,35]	Moderate confidence	3 studies with no or very minor methodological limitations, relevance, coherence and adequacy.

Discussion

This systematic review and thematic synthesis summarize how HCPs advocate for patient safety across diverse healthcare settings worldwide. Key PA themes included interprofessional collaboration, risk prevention, education and informed consent, safety, and medication management. The data presented describes HCPs’ advocacy practices when promoting patient safety in diverse healthcare settings worldwide.

Interprofessional Collaboration in Patient Advocacy

Interprofessional collaboration was the most frequently identified theme, present in 12 of 16 included studies, suggesting it is a central mechanism through which HCPs enact PA to promote patient safety. This included working with multidisciplinary teams, raising concerns or disagreements about patients’ conditions and treatment plans, and maintaining thorough documentation to ensure continuous promotion of patient safety. Evidence from the literature indicates that HCPs in primary and secondary care settings agree that positive interprofessional relationships enhance PA and lead to improved patient outcomes [38].

For instance, Kucukarslan et al. (2003) reported that collaborating with pharmacists in intensive care settings reduced preventable adverse drug events (ADEs) by 78% (from 26.5 to 5.7 ADEs per 1000 patient-days), and shortened hospital stays by 1.4 days [39]. These findings highlight that effective collaboration enhances patient safety and improves efficiency of care. Collaboration and coordination among HCPs are essential for promoting integration across different levels of care. Strong communication channels not only strengthen these collaborative processes but also facilitate the transfer of important knowledge and continuity of care between secondary and primary care settings.

By contrast, another qualitative study found that main barriers to establishing seamless care for patients included lack of trust among HCPs, a desire to control patient care in the hospital, lack of role clarity, and practical issues such as incompatibility of medical records systems [40]. Addressing these obstacles is essential for creating collaborative systems where PA can flourish.

Advocacy Through Risk Prevention

Strategies aimed at reducing patient risks include ongoing professional education, guidelines compliance, vigilant monitoring, problem-solving, early discharge planning, and balancing patient autonomy with safety. Training has been found to be a critical enabler of patient safety, enhancing knowledge and perceived quality of care [41]. For example, implementation of the TeamSTEPPS program resulted in more than 50% improvement in patient safety culture scores from 98.42 ± 38.0 at baseline to 150.50 ± 10.7 post training and 151.1 ± 14.5 at two months, indicating sustained knowledge retention and positive cultural change [42]. Such programs show how structured, team-based training can strengthen advocacy and reduce harm.

Patient Advocacy Through Education and Informed Consent 

Advocacy through education and informed consent emerged as an important contributor to patient safety. Respecting patients’ rights to receive clear information, participate in shared decision-making, and feel supported throughout their care journey is fundamental to high-quality clinical practice. This includes ensuring that patients understand treatment options, procedures, associated risks, and responsibilities related to post-discharge at-home care. Examples of advocacy-driven communication include obtaining informed consent before procedures, providing written post-discharge instructions, and implementing needs-based education [43,44]. Structured education has been shown to improve patient outcomes. For example, Ndosi et al. (2016) demonstrated that needs-based education significantly improved self-efficacy and symptom burden over time [45]. Such findings reinforce that patient education is an ethical obligation as well as an effective safety intervention.

Safety-Oriented Patient Advocacy

Safety-oriented advocacy was reflected in how HCPs allocated limited resources toward the most urgent clinical needs, occasionally requiring trade-offs with patient comfort to achieve life-saving or clinically optimal outcomes. Ryynanen et al. (1998) reported that high-technology surgical interventions for life-threatening conditions and pain relief for terminally ill patients were consistently ranked as the highest priorities, while cosmetic procedures were considered lowest [46]. Similarly, Becker et al. (2015) found that 94.5% of patients presenting to the emergency room were discharged the same day, with 91.3% classified as low priority, while high-priority cases had significantly higher hospitalization rates, longer lengths of stay, and greater mortality [47]. These findings illustrate how safety-oriented advocacy relied on clinical prioritization to guide effective resource allocation, ensuring that patients with the most critical needs receive timely, safe, and effective care.

Advocacy in Medication Management and Polypharmacy

Advocacy in medication management and polypharmacy required vigilant monitoring, coordination, and patient engagement to ensure safe and effective treatment. Common strategies included conducting frequent medication reviews, clarifying prescriptions with colleagues, and educating patients on correct medication use. McIntosh et al. (2018) reported that patients aged ≥ 50 years taking five or more medications were most frequently identified for review, with structured medication discussions reducing the risk of adverse events [48]. Similarly, Gerard et al. (2020) found 500 (2.1 ± 3.9 per patient) discrepancies in 237 patients’ medical records, with 53.6% experiencing at least one inconsistency [49]. Pharmacists also identified 860 drug therapy problems (3.6 per patient), with 22.6% involving patients remaining untreated for a diagnosed indication [49]. Proactive advocacy-oriented medication management practices support error prevention, treatment adherence, and patient safety.

Overall, evidence suggests that PA practices contribute to improved patient safety and quality of care [12]. The results of this systematic review reinforce the importance of integrating PA into organizational culture with training, collaboration, and support structures that enable HCPs to voice concerns and act on behalf of patients. Addressing barriers such as time pressures, unclear roles, and system incompatibility remain essential [1,13,50]. Expanding advocacy training that integrates patient safety competencies may enhance HCPs’ capacity to translate advocacy skills into demonstrable improvements in clinical safety and quality of care.

Strengths and Limitations

This review possesses several notable strengths. To our knowledge, it is the first thematic synthesis to systematically examine how HCPs enact PA practices to promote patient safety across multiple healthcare professions and international contexts. The inclusion of studies from 13 countries provides a global perspective and offers insights into cross-cultural and systemic variations in how advocacy is enacted in clinical practice. A systematic and transparent search and screening process, reported in accordance with PRISMA guidelines and informed by the PERSiST framework for qualitative synthesis, strengthen methodological credibility and replicability. Furthermore, the inclusion of qualitative studies offers a nuanced understanding of how practicing HCPs understand and enact PA in real-world clinical settings, adding contextual factors that may not be captured through quantitative measures alone. By integrating evidence from diverse settings and methodologies, this review provides a foundation for future research and the development of evidence-informed interventions that position advocacy as a mechanism for improving patient safety.

This review also has several limitations. First, physicians were underrepresented in the included studies, limiting insight into their perspectives on PA and safety, and most participants were female. Most studies were qualitative, which provided rich understanding of complex interactions and informed potential practice and policy changes. However, these designs typically involved small samples and may be subject to research bias. The two cross-sectional studies included had methodological weaknesses: one lacked detail about the type of data collected, and neither reported power analyses to justify sample size, raising concerns about statistical validity.

Another limitation is the variability of study settings. Although the included studies spanned 13 countries, enhancing diversity, cultural and systemic differences restrict generalizability to other regions or healthcare systems. Additionally, only three databases were searched, and grey literature was excluded, so relevant unpublished or non-indexed studies may have been missed, and the possibility of publication bias cannot be ruled out. Furthermore, two records were identified through preliminary supplementary searching conducted prior to the formal database search. While both records were assessed using the same eligibility criteria and verified as peer-reviewed publications, full reproducibility of this identification step cannot be guaranteed given its preliminary nature.

Future Research

Future research should include a broader range of HCPs, particularly physicians and technicians, who play key roles in patient care but were largely absent from current studies. Quantitative and mixed-methods studies are needed to complement qualitative insights and to capture patient and caregiver perspectives on advocacy practices across different care settings and facility types. Research that includes multicultural and geographically diverse populations would help clarify how cultural context shapes advocacy behaviors. Finally, future work could examine the role of provider attributes such as emotional intelligence, moral intelligence, and PA engagement, which may influence how advocacy is enacted and perceived in clinical practice. Interventional studies, including training programs or organizational initiatives, could provide stronger evidence for strategies that promote effective PA and its effect on safety outcomes.

## Conclusions

This review synthesized evidence describing PA as a core mechanism for advancing patient safety and identified five key themes that explain how HCPs support patient safety across healthcare settings: interprofessional collaboration, risk prevention, education and informed consent, safety-oriented PA, and medication management and polypharmacy. Together, these themes highlight how HCPs promote safety through collaborative decision-making, proactive risk mitigation, patient engagement, clinical prioritization, and vigilant medication management, emphasizing the importance of communication, ethical decision-making, and clinical vigilance in minimizing risks and ensuring high-quality care. 

The findings highlight the need for ongoing professional training to build advocacy-related skills, effective communication, and ethical decision-making. Education curricula should intentionally embed advocacy principles within core coursework and competency assessment, while healthcare organizations should align policies, training, and culture to support clinicians in acting as advocates for patient safety. Implementing these strategies may help reduce preventable harm, MEs, and SEs, ultimately advancing safer, more reliable, and patient-centered care delivery.
